# 

**Published:** 1857-09

**Authors:** 


					﻿PUBLISHED MONTHLY, AT S3 PER ANNUM IN ADVANCE.
i
—
1
3
s
Q
C
Z
g
5
1
2
s
z
z
n
T.
Q
c
2
z
wt
z
o
2
?
>-*
I.
K
rk
X
o
.V l' L A N T A
MIDICAL 111 SlllliH IL
I 0 V B ■ A £ ® _■
EDITED BY
JOSEPH P. LOGAN, M. D.,
Professor of Physiology and General Pathology in the Atlanta Medical College.
a N D
W. F. WESTMORELAND, M. D.,
Professor of the Principles axt> Practice of Surgery ix the Atlanta Medical College,
Pax et scientia, sed veritas sine timore.
Vol. hi. SEPTEMBER, 1857. No. 1.
ATLANTA, GEORGIA:
G. P. EDDY & CO., PUBLISHERS AND PROPRIETORS.
1 8 5 7.
EACH NUMBER CONTAINS SIXTY-FOUR PAGES,.
o
X
5
X
-5
X
6
■9
y
**!
5.
2
n?
r*
>
x
co
X
o
-5
'JD
				

